# An observational study to evaluate factors predicting survival in patients of non-small cell lung cancer with poor performance status in resource-constrained settings

**DOI:** 10.3332/ecancer.2021.1274

**Published:** 2021-08-05

**Authors:** Akhil Kapoor, Vanita Noronha, Amit Joshi, Vijay M Patil, Nandini Menon, Rajesh Bollam, Vikas Talreja, Supriya Goud, Sucheta More, Leena Solanki, Srushti Shah, Anuradha Chougule, Abhishek Mahajan, Kumar Prabhash

**Affiliations:** 1 Department of Medical Oncology, Mahamana Pandit Madan Mohan Malviya Cancer Centre & Homi Bhabha Cancer Hospital, Tata Memorial Centre, Varanasi, Uttar Pradesh 221005 India; 2 Department of Medical Oncology, Tata Memorial Hospital, Tata Memorial Centre, Homi Bhabha National Institute (HBNI), Mumbai 400012 India; 3 Department of Molecular Oncology, Tata Memorial Hospital, Tata Memorial Centre, Homi Bhabha National Institute (HBNI), Mumbai 400012 India; 4 Department of Radiodiagnosis, Tata Memorial Hospital, Tata Memorial Centre, Homi Bhabha National Institute (HBNI), Mumbai 400012 India; † The first two authors contributed equally to the manuscript.; a https://orcid.org/0000-0001-6006-2631; b https://orcid.org/0000-0001-6716-5238

**Keywords:** poor performance status, non-small cell lung carcinoma, oral tyrosine kinase inhibitors, EGFR mutation

## Abstract

**Background:**

A significant proportion of non-small cell lung cancer (NSCLC) patients present with poor performance status (PS) at baseline are almost always excluded from the clinical trials leading to availability of only limited data in this subgroup.

**Patients and methods:**

This was an observational single institutional study. The eligibility criteria for inclusion were a histologic or cytologic diagnosis of advanced NSCLC and Eastern Cooperative Oncology Group PS 3 or 4. All patients coming between June 2015 and December 2018 were evaluated for inclusion in this study.

**Results:**

A total of 245 patients were enrolled in the study. The median age of the patients was 63 years (range 25–89), 142 (58%) were male, 196 (80%) had adenocarcinoma histology and 192 (78.4%) has PS 3 while rest (21.6%) had PS 4. Out of 245 patients, 192 (78.4%) received oral tyrosine kinase inhibitors (TKI) and supportive care, 45 (18.4%) received supportive care alone, while 8 (3.2%) patients received chemotherapy along with supportive care. Median overall survival (OS) was 3 months (95% CI: 1.8–4.2) in patients who received oral TKI versus 1 month (1.0–2.9) in patients who received supportive care alone (log-rank *p* = 0.013). The median OS for epidermal growth factor receptor (EGFR) mutant patients who received oral TKI was 12 months (95% CI: 7.7–16.3), while it was 3 months (95% CI: 1.5–4.5) for patients who were EGFR wild-type and received TKI on compassionate basis (HR = 0.50; 95% CI: 0.32–0.77; *p* = 0.001).

**Conclusions:**

The use of oral TKI on a compassionate basis led to improvement in survival in the overall cohort of the patients; this was principally driven by EGFR-mutated patients.

## Introduction

A significant proportion of non-small cell lung cancer (NSCLC) patients present with poor performance status (PS) at baseline. Lilenbaum *et al* [1] reported the prevalence of poor PS to be 34% when estimated by providers, while it was as high as 48% when estimated by patients themselves. A recent review by Friedlaender *et al* [2] found that 35% of NSCLC patients have PS 2 at diagnosis. The poor PS patients (Eastern Cooperative Oncology Group [ECOG] PS 3–4) are almost always excluded from the clinical trials leading to availability of only limited data in this subgroup [3]. An analysis from our group which highlighted the reasons for management of epidermal growth factor receptor (EGFR) mutant patients outside the clinical trial found that compromised ECOG PS >2 was the major reason (36.9%) for ineligibility of patients in a clinical trial [4]. Thus, it is clear that poor PS patients form a large chunk of new NSCLC cases in the real-world scenario and are under-represented in trials.

Immunotherapy with or without chemotherapy is the current standard of care for patients with advanced NSCLC with no actionable mutations. Oral tyrosine kinase inhibitors (TKI) are standard treatment for patients with driver mutations. EGFR mutations are present in about one-third of adenocarcinomas of the lung in patients of Asian origin [4]. Due to relative ease of administration and acceptable toxicity profile of EGFR inhibitors, the trials of these drugs included patients with PS 2 [5]. Erlotinib was considered a suitable option as second or third treatment for patients with advanced NSCLC unselected for EGFR mutation [6]. This study had shown improved overall survival (OS) with erlotinib 6.7 months versus 4.7 months, HR = 0.70 and *p* < 0.001. An important point to be noted is that around half of the patients were ≥60 years and one-third had PS 2–3. Another analysis of the same study found that older patients (≥70 years) treated with erlotinib benefited the same as young patients, albeit with greater toxicities [7]. After these studies, erlotinib was also tried in chemotherapy-naive older patients (≥70 years) with advanced NSCLC, unselected for EGFR and found an OS of 10.9 months (95% CI: 7.8–14.6 months) [8]. Of note, 10% of the patients had PS 2 at baseline and those patients who developed treatment-related rash (79%) had significantly better progression-free survival (PFS) and OS, with a median OS of 14.3 months versus 4.2 months.

Considering the limited data in poor PS patients from clinical trials, real-life data become useful in decision-making. Thus, we conducted an observational study to find out various aspects of these poor PS patients irrespective of the treatment offered which may help clinicians in the decision-making process in the clinic.

## Patients and methods

### Trial design and conduct

This was an observational single institutional study. The study was approved by the institutional ethics committee. All patients provided written informed consent prior to participating in the study. The study is registered with the Clinical Trials Registry of India (CTRI/2014/11/005216). The study was conducted in accordance with the guidelines for good clinical practice – ICH E6(R2), Declaration of Helsinki and Indian Council of Medical Research guidelines. This research did not receive any specific grant from funding agencies in the public, commercial or not-for-profit sectors.

### Participants

The eligibility criteria for inclusion were a histologic or cytologic diagnosis of advanced NSCLC and ECOG PS 3 or 4. The patients should have been chemotherapy-naïve, older than 18 years and able to take oral medications. Patients were excluded if they had received any cancer-directed therapy previously.

### Interventions

Patients were evaluated in the multidisciplinary thoracic oncology disease management tumour board; investigations and therapy were decided by the treating team. Subsequently, they were evaluated in the Medical Oncology department of thoracic disease management group for decision and treatment options. There was no separate intervention for this study purpose. No formal sample size calculation was carried out. All patients who presented between June 2015 and December 2018 were evaluated for inclusion into this study. For patients with poor PS, only EGFR and Anaplastic Lymphoma Kinase (ALK) were carried out as a part of the institutional protocol.

Adverse events during treatment were documented and graded using the common terminology criteria for adverse events, version 4.02. OS was calculated from the date of starting supportive care to the date of death. Patients who were still alive were censored on the date of last contact. All patients underwent baseline radiologic imaging using computerizsed tomography (CT) scan/positron emission tomography (PET) CT scan and magnetic resonance imaging/CT brain (optional). Patients who were started on oral TKI or chemotherapy were advised response CT scans every 2–3 months. The imaging scans were reported by experienced oncologic radiologists at the institution, as per the institutional practice. At each visit of the patient to the hospital, the database was updated. If the patient did not return to the hospital for follow-up, we attempted to contact the patient telephonically.

The database was maintained in an Microsoft Excel format. The details entered included the patient demographics, disease-related details, investigations, treatment planned, treatment delivered, symptoms, toxicity, progression and survival information. Demographic details were calculated by descriptive analysis. Toxicity data were presented with absolute numbers and simple percentages. Response rate was calculated using simple percentages. The Kaplan–Meier method was used to calculate the overall survival and log-rank test was used to analyse the various clinicopathological factors for their effect on survival. Cox regression analysis was used to carry out univariate and multivariate analyses. Statistical Package for the Social Sciences version 20.0 was used for all statistical calculations.

## Results

**Figure 1.**
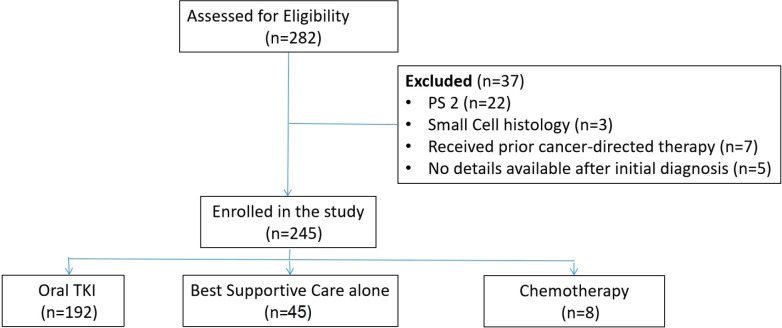
Flow diagram of the study.

A total of 245 patients were enrolled in the study, the reasons for exclusion are shown in Figure 1. Table 1 depicts the baseline characteristics of the studied patients. The median age of the patients was 63 years (range 25–89), 142 (58%) were male, 196 (80%) had adenocarcinoma histology, 192 (78.4%) has PS 3 while the rest (21.6%) had PS 4. Smoking history was present in 110 (44.9%) patients. In 28 (11.4%) patients, biopsy could not be obtained due to poor PS; 21 (8.6%) patients had cytology for diagnosis of cancer, while 7 (2.8%) underwent fine-needle aspiration cytology (FNAC). Out of 245 patients, 10 (4%) had stage III disease, while 70 (28.5%) had stage IVA disease and 165 (67.3%) had stage IVB disease. Out of 245 patients, 132 (53.8%) patients had comorbidities with diabetes (16.3%), with hypertension (13.5%) being the most common. Cough was the most common symptom being present in 64.9%, followed by dyspnoea (48.6%) and chest pain (26.5%).

Out of 245 patients enrolled in the study, 192 (78.4%) received oral TKI and supportive care, 45 (18.4%) received supportive care alone, while 8 (3.2%) patients received chemotherapy along with supportive care. Median OS was 3 months (95% CI: 1.8–4.2) in patients who received oral TKI versus 1 month (1.0–2.9) in patients who received supportive care alone (log-rank *p* = 0.013). Patients’ survival at 6 months in TKI group was 32.7% (SD 3.5) versus 14.9% (SD 5.6) in the supportive care alone group. The data for toxicities of TKI were available in 40 (20.8%) patients and it was well tolerated in majority of the patients with all grade rash in 27 (67.5%) patients; grade 3 rash occurred in 3 (7.5%) patients, grade 3 diarrhoea in 1 (2.5%) patient and grade 3 transaminitis in 2 (5%) patients. Out of the 8 patients who received chemotherapy, 4 (50%) developed grade 3/4 anaemia, 1 (12.5%) had grade 3 febrile neutropenia and 3 (37.5%) patients developed grade 3 thrombocytopenia.

**Figure 2.**
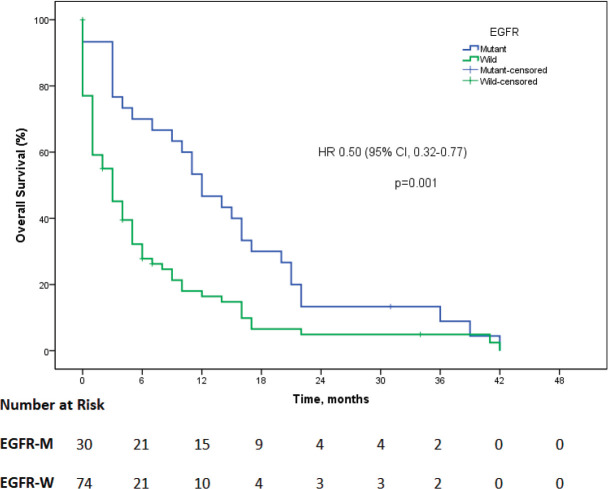
The Kaplan–Meier survival curve showing overall survival as the EGFR mutation status in patients who were started on TKI on compassionate basis (EGFR-M = EGFR mutant, EGFR-W = EGFR wild).

The data for EGFR mutation status were available for 119 (48.6%) patients, out of which 31 (26.2%) were positive for EGFR mutation. Exon 19 mutation was identified in 20 (34.5%), exon 21 in 9 (29%), while complex mutations were found in 2 (6.4%) of the patients. Out of 31 EGFR-mutated patients, 2 (6.4%) had squamous cell carcinoma, while the rest had adenocarcinoma. EGFR testing was carried out in 102 patients with adenocarcinoma histology and 17 patients with squamous cell carcinoma (SCC). Thus, the EGFR mutation rate was 28.4% in adenocarcinoma and 11.76% in SCC. Among the patients in whom EGFR status was available, 104 patients were started on oral TKI on compassionate basis without waiting for the EGFR report. The oral TKI used was gefitinib in 90 (86.5%) patients, while the rest received erlotinib. The median OS for EGFR mutant patients who received oral TKI was 12 months (95% CI: 7.7–16.3), while it was 3 months (95% CI: 1.5–4.5) for patients who were EGFR wild-type and received TKI on compassionate basis (HR = 0.50, 95% CI: 0.32–0.77, *p* = 0.001; Figure 2). ALK testing was carried out in 105 patients, out of which it came as mutated in 11 (10.5%) patients, and 4 (36.3%) of these patients were started on crizotinib.

ECOG PS, smoking, EGFR mutation status and use of EGFR TKI on compassionate basis came as significant factors on univariate analysis for OS (Table 2). There was no difference in OS on the basis of histology, age, gender, comorbidities and stage. The patients with ECOG PS 3 had better survival (median OS: 3 months; 95% CI: 1.8–4.0) than patients with PS 4 (median OS: 1 month; 95% CI: 0.2–1.7; *p* = 0.011). Similarly, OS was significantly better in patients who were non-smokers (median OS: 4 months; 95% CI: 2.2–5.7) when compared to smokers (median OS: 1 month; 95% CI: 0–2.0; *p* = 0.001). On multivariate analysis, smoking and EGFR mutation status were the significant factors affecting OS (Table 2). Among EGFR-mutated patients, the median OS was 16 months (95% CI: 10.6–21.4) for exon 19 deletion, while it was 11 months (95% CI: 6.6–15.4; *p* = 0.08) for patients with other EGFR mutations.

## Discussion

This is an observation study on NSCLC patients with PS 3–4 which is very uncommon in the existing literature. This study showed that patients receiving oral TKI on compassionate basis for patients with baseline poor ECOG PS had better survival when compared to the group not receiving oral TKI. There was doubling of OS at 6 months with the use of oral TKI (14.9% versus 32.7%, log-rank *p* = 0.013). The TOPICAL study, which randomised 770 patients unsuitable for chemotherapy to receive erlotinib versus placebo, found that the median OS did not differ between the two treatment groups; however, patients developing rash with erlotinib during the first month of use had significantly better OS versus placebo (HR = 0.76; 95% CI: 0·63–0·92; *p* = 0.005) [9]. This suggests that this clinical selection was one way to provide this effective treatment to these patients. It should be noted that the incidence of EGFR mutation in this study was relatively low (7%), whereas it was 26% in our study. This may explain the benefit in terms of OS for oral TKI use on compassionate basis in our study. The EGFR mutation rate was 28.4% in adenocarcinoma and 11.8% in SCC. These data are in congruence with previously published studies from our institute [10, 11].

The National Comprehensive Cancer Network (NCCN) recommends TKI for patients with driver mutations in NSCLC patients. However, an important limitation of this approach remains that we need to wait till the report is available. This will take approximately 2–3 weeks for report to be available as in our settings. In a significant proportion of these patients, biopsy was not feasible. One of the solutions is having liquid biopsy (especially by droplet digital polymerase chain reaction (ddPCR)), but its utility is limited by its availability and the sensitivity of this test.

In our study, only PS 3–4 patients were enrolled. To our knowledge, this is the only study dedicated exclusively to PS 3–4 patients. In the TOPICAL study, PS 2 patients were also included. Despite this, there was significant OS benefit in our study. Also, there were 58.7% patients who were ≥60 years in this study which is clearly higher than previous data from our institute (24.3%) [12]. This points towards higher chances of presenting with poor PS in older patients. However, it is reassuring that a pooled analysis from the same authors concluded that EGFR TKI led to a similar survival in patients aged 60 years or older when compared to younger patients, except for a higher incidence of diarrhoea in older patients [12].

In a randomised phase II study by Chen *et al* [13], erlotinib was compared with vinorelbine in chemotherapy-naive, EGFR-unselected patients aged ≥70 years, with 23% of the patients having PS 2–3 [13]. The median OS was similar in the two groups (11.7 months for erlotinib versus 9.3 months for vinorelbine, *p* = 0.70). In our study, only eight (3.2%) patients received chemotherapy; hence, such comparison could not be made. In the IFCT-0301 study, EGFR-unselected patients with PS 2–3 were randomised to first-line gefitinib, gemcitabine or docetaxel [14]. The survival rates were similar in all groups with higher toxicities in docetaxel group. All these studies point towards the option of using oral EGFR TKI in unselected patients with poor PS at baseline. In our study, the use of EGFR TKI was a significant factor affecting the OS in the univariate analysis along with ECOG PS and smoking history. It is well established that smokers have poorer progression-free survival when receiving first-line EGFR TKI when compared with never-smokers [12, 15]. The mutation burden in smokers is estimated to be at least 10 times higher in lung adenocarcinoma patients when compared to never-smokers [16]. These mutations can occur in DNA mismatch repair genes resulting in secondary resistance to EGFR TKI besides activating various bypass pathways [16]. In our study, HR for smokers was 1.52 (95% CI: 1.16–1.98; *p* = 0.002) when compared to never smokers and this was significant on multivariate analysis also signifying that smoking is an independent prognostic factor in the study patients. It should be noted that this study evaluated treatment-naive patients with baseline ECOG PS 3–4, while most of the previous studies have included patients with PS 2–3 or pre-treated patients with PS 3–4 [17]. At the same time, PS continues to be an important prognostic factor for OS even in EGFR-mutated patients. In a study by Yao *et al* [18], patients with EGFR-mutated NSCLC treated with first-line gefitinib had a much poorer median OS for PS ≥2 (14 months; 95% CI: 8.0–20.0) when compared to overall median OS of 26.9 months (95% CI: 21.2–32.5; *p* < 0.001) [18].

This study provides real-world data on the benefit of using oral TKI on a compassionate basis in poor PS patients at baseline. It led to significant improvement in OS primarily driven by EGFR-mutated patients. This approach is useful in Asian countries where the incidence of EGFR mutation is higher when compared to the West. The present study suffers from some important limitations. This study was not a randomised one and the toxicity data were available only in a limited number of patients. Besides, EGFR testing was possible in only 50% of the patients because the patients were deemed unfit for any cancer-directed therapy by the treating physician. Also, the ideal approach would have been rapid testing of the EGFR and ALK status and offering immunotherapy. However, this is not possible in constrained resources settings in the real world. The authors would like to highlight that this study was carried out till 2018, when the use of PDL1 testing and other mutations were rare. An important strength of the study being inclusion of only PS 3–4 patients for which very limited data are available.

## Conclusion

The use of oral TKI on a compassionate basis led to improvement in survival in the overall cohort of the patients; this was principally driven by EGFR-mutated patients which formed around 26% of the tested patients which is in congruence with previously reported EGFR mutation rates.

## Availability of data and material

The raw data can be made available on appropriate request making sure the anonymity of the study participants.

## Authors’ contributions

The study conception and design was performed by Vanita Noronha, Amit Joshi and Kumar Prabhash. Data collection was performed by Vijay M Patil, Nandini Menon, Rajesh Bollam, Vikas Talreja, Supriya Goud, Sucheta More, Leena Solanki, Srushti Shah, Anuradha Chougule and Abhishek Mahajan. Data analysis was performed by Akhil Kapoor, Vanita Noronha, Amit Joshi and Kumar Prabhash. The first draft of the manuscript was written by Akhil Kapoor and all authors commented on previous versions of the manuscript. All authors read and approved the final manuscript and guaranteed integrity of the entire study.

## Conflicts of interest

Dr Vanita Noronha reports grants from Dr Reddy’s Laboratories Inc., grants from Amgen, grants from Sanofi/Aventis, grants from AstraZeneca and outside the submitted work. All grants were paid to the institution. Dr Kumar Prabhash reports grants from Dr Reddy’s Laboratories Inc., grants from Fresenius Kabi India Pvt Ltd, grants from Alkem Laboratories, grants from Natco Pharma Ltd., grants from BDR Pharmaceuticals India Pvt. Ltd., grants from Roche Holding AG and outside the submitted work. All grants were paid to the institution. All other authors have nothing to disclose.

## Funding

The authors did not receive any funding for conducting this study.

## Figures and Tables

**Table 1.** Baseline characteristics of the patients.

Characteristics	Number (Percentage)
Age	Median: 63 years
	Range: 25–89 years
Gender	
Male	142 (58.0)
Female	103 (42.0)
Histology	
Adenocarcinoma	196 (80.0)
Squamous	47 (19.2)
Others	2 (0.8)
ECOG PS	
3	192 (78.4)
4	53 (21.6)
Smoking	
Ever smoker	110 (44.9)
Never smoker	135 (55.1)
Stage	
III	10 (4.0)
IVA	70 (28.5)
IVB	165 (67.3)
Comorbidities	
None	113 (46.2)
Hypertension	33 (13.5)
Diabetes mellitus	40 (16.3)
COPD or emphysema	28 (11.4)
Prior tuberculosis	7 (2.9)
Others	4 (1.6)
Multiple comorbidities (>1)	20 (8.1)
EGFR mutation^a^	
Exon 19 mutation	20 (16.8)
Exon 21 mutation	9 (7.6)
Complex mutation	2 (1.7)
Wild	88 (73.9)
Brain metastasis	
Yes	38 (15.5)
No	207 (84.5)

a Tested in 119 patients

**Table 2.** Univariate and multivariate analyses of various factors affecting the overall survival by Cox regression analysis.

Characteristics	*N* (%)	Univariate HR (95% CI)	*p* value	Multivariate HR (95% CI)	*p* value
Age (years)					
<60	101 (41.2)	Ref	0.862		
≥60	144 (58.7)	0.98 (0.75–1.27)			
Gender					
Male	142 (58.0)	Ref	0.109		
Female	103 (42.0)	0.80 (0.61–1.05)			
ECOG PS					
3	192 (78.4)	Ref	0.023	Ref	0.408
4	53 (21.6)	1.46 (1.05–2.00)		1.16 (0.82–1.64)	
Smoking					
Never smoker	110 (44.9)	Ref	0.002	Ref	0.014
Ever smoker	135 (55.1)	1.52 (1.16–1.98)		1.40 (1.07–1.83)	
Comorbidities					
None	113 (46.2)	Ref	0.439		
Present	132 (53.8)	0.90 (0.69–1.17)			
Histology					
Adenocarcinoma	196 (80.0)	Ref	0.623		
Squamous	47 (19.2)	1.09 (0.78–1.51)			
Stage					
III	10 (4.0)	Ref			
IVA	70 (28.5)	1.04 (0.52–2.10)	0.925		
IVB	165 (67.3)	0.86 (0.46–1.65)	0.658		
EGFR TKI received^a^					
No	44 (18.6)	Ref	0.027	Ref	0.941
Yes	193 (81.4)	0.68 (0.48–0.96)		1.07 (0.72–1.42)	
EGFR mutation					
Mutated	31 (12.6)	0.36 (0.24–0.54)	<0.001	0.39 (0.25–0.60)	<0.001
Wild-type	87 (35.5)	0.62 (0.46–0.82)	0.001	0.65 (0.48–0.88)	0.005
Unknown	124 (50.6)	Ref		Ref	
Brain metastasis					
No	207 (84.5)	0.82 (0.57–1.19)	0.315		
Yes	38 (15.5)	Ref			

a Eight patients who received chemotherapy as first-line therapy were excluded from this analysis
